# Microbiota-derived metabolites in regulating the development and physiology of *Caenorhabditis elegans*

**DOI:** 10.3389/fmicb.2023.1035582

**Published:** 2023-02-28

**Authors:** Min Feng, Baizhen Gao, L. Rene Garcia, Qing Sun

**Affiliations:** ^1^Department of Chemical Engineering, Texas A&M University, College Station, TX, United States; ^2^Department of Biology, Texas A&M University, College Station, TX, United States

**Keywords:** bacterial metabolite, animal development, physiology, host-microbiota interaction, *Caenorhabditis elegans*

## Abstract

Microbiota consist of microorganisms that provide essential health benefits and contribute to the animal’s physiological homeostasis. Microbiota-derived metabolites are crucial mediators in regulating host development, system homeostasis, and overall fitness. In this review, by focusing on the animal model *Caenorhabditis elegans*, we summarize key microbial metabolites and their molecular mechanisms that affect animal development. We also provide, from a bacterial perspective, an overview of host-microbiota interaction networks used for maintaining host physiological homeostasis. Moreover, we discuss applicable methodologies for profiling new bacterial metabolites that modulate host developmental signaling pathways. Microbiota-derived metabolites have the potential to be diagnostic biomarkers for diseases, as well as promising targets for engineering therapeutic interventions against animal developmental or health-related defects.

## 1. Introduction

Animal growth and development are fundamental processes controlled by genetic factors, nutrient intake, as well as interactions with environments and other organisms. As a model organism, *Caenorhabditis elegans* has been extensively used for investigating fundamental signaling pathways because of its genetic homology to humans (Shapira, 2017; Zhang et al., 2017). *Caenorhabditis elegans* is a bacterivore grown in the laboratory using *Escherichia coli* OP50 as a standard food source, the larvae develop through four stages (L1-L4) before molting into reproductive adults. The phenotypes of worms, fed with different bacterial isolates, can be screened for their responses to chemical perturbations. In past decades, multiple conserved regulatory pathways have been characterized using this model organism. For instance, the transformation growth factor-β (TGF-β) superfamily are found to be essential regulators of developmental arrest, body-size determination, male copulatory structures, and axonal guidance in *C. elegans* development (Patterson and Padgett, 2000). In addition, small-molecule ligands of nuclear hormone receptors (NHRs), such as DAF-12, govern the transcriptional regulation of *C. elegans* larval development (Mahanti et al., 2014). Additionally, the heterochronic genes of *C. elegans* encode an RNA-mediated gene regulatory system that controls the temporal component of cell fates associated with arrested development (Ambros and Moss, 1994).

Multicellular organisms live in complex environments and must respond to environmental changes, as well as differences in nutrient availability. As a result, metabolic networks change to meet cellular and metabolic requirements. In regards to changes in nutrient availability and other environmental cues, the Target of Rapamycin (TOR), a serine/threonine kinase, regulates growth and development by modulating protein synthesis, autophagy, and multiple other cellular processes (Long et al., 2002; Blackwell et al., 2019). The insulin/insulin-like growth factor (IGF) signaling pathway is another major pathway that regulates diapause (developmental quiescence) in response to a depletion of nutrients (Narasimhan et al., 2009). In presence of food, an insulin receptor family member, DAF-2, regulates glycolytic and fat metabolism to promote reproductive development into adulthood in *C. elegans* (Kimura et al., 1997). In addition, NHRs are small-molecule ligands that sense signals from metabolic and environmental conditions, and govern the transcriptional regulation of *C. elegans* larval development (Pardee et al., 2011; MacNeil et al., 2013; Mahanti et al., 2014). Interestingly, it has been uncovered that microbiota can interact with the host by leveraging these growth-related signaling, and eventually impact host development.

As crucial mediators of diet-induced host–microbe interactions, metabolites derived from microbiota can influence an animal’s developmental process through conserved metabolic pathways. Microbial metabolites contribute to various developmental and physiological processes, such as post-embryotic development, energy metabolism, etiopathogenesis of diseases, tissue adaption, as well as the differentiation of the immune system, providing a considerable boost to the host’s biochemical and metabolic capabilities (The Human Microbiome Project Consortium, 2012). Emerging studies have discovered specific metabolites function as signaling molecules to regulate animal development and maintain metabolic balance. In this review, we summarized microbial metabolites and their roles in regulating animal growth and development. Furthermore, we discussed the methods that are driving new advancements in microbial metabolites identification, as well as their potential in future bacteria-host interaction research.

## 2. Microbial metabolites regulate host development and physiology

**Figure 1:**
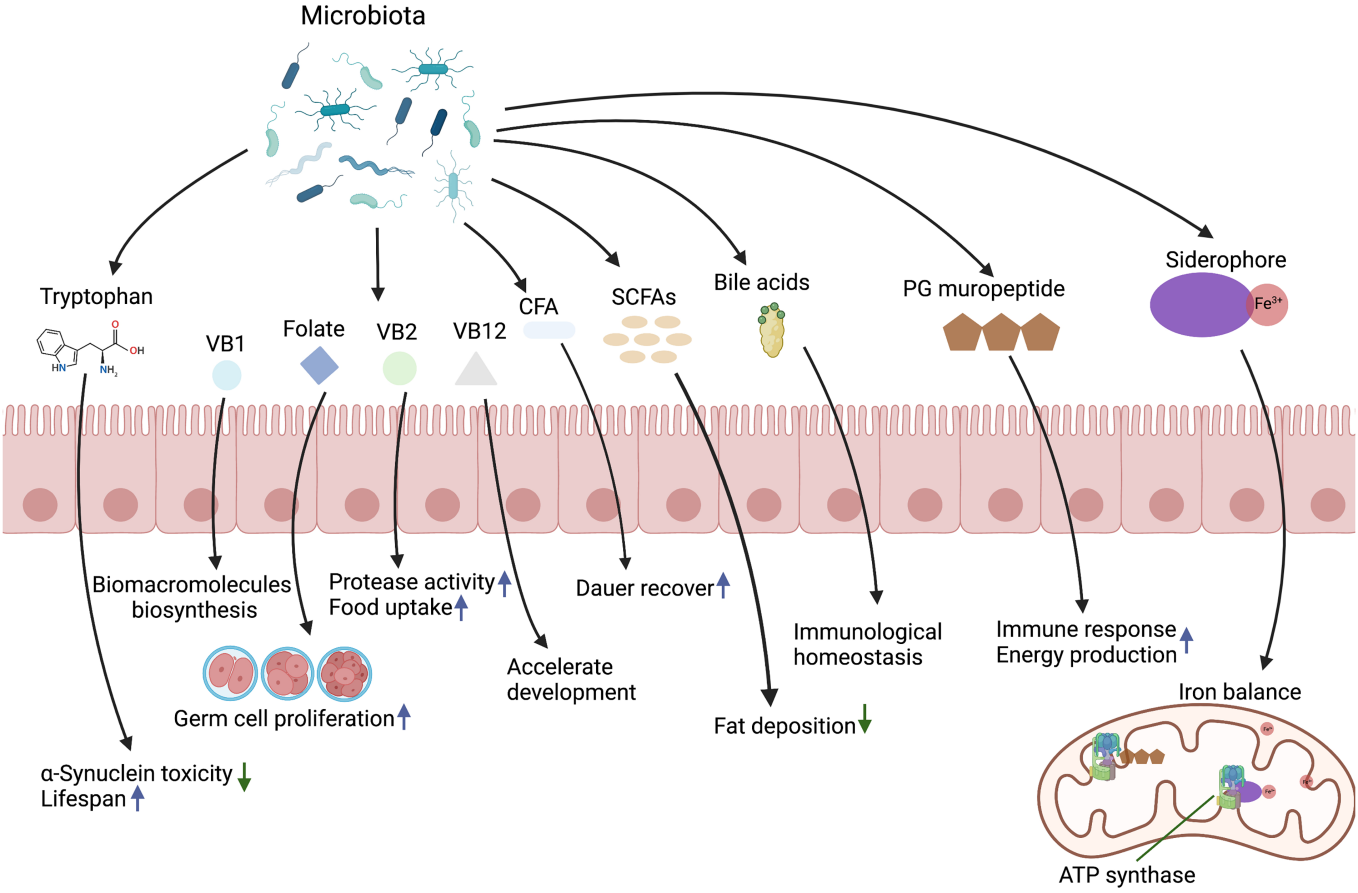
Effects of microbiota-derived metabolites on host development and physiology.

Microbial metabolites are required for several aspects of animal physiology including development, differentiation of intestinal epithelium, immune system activation, energy production, and systemic homeostasis. These metabolites provide a diverse range of biochemical and metabolic activities to complement host physiology. In this section, we will review the key microbial molecules and associated mechanisms in regulating host development and physiology (Figure 1).

### 2.1. Vitamins

Vitamins are organic substances essential for the proper development of animals. They are primarily obtained from dietary sources, either due to the fact that animals cannot synthesize them or cannot produce sufficient amounts (Combs and McClung, 2016). As the key participants in the digestion of food, intestinal bacteria are responsible for the synthesis of most water -soluble vitamins from the diet (Said, 2011). In *C. elegans*, established approaches have been developed to induce certain vitamin deficiencies or introduce extra vitamins, making it an ideal organism to study the effects of microbial derived vitamins on life history traits.

Folates, a B-group vitamin, is pivotal to DNA replication, nucleic acid synthesis, methionine regeneration, and cell division. Folate is incapable of being synthesized by animals and therefore needs to be obtained from the animal’s diet or microbiota. The bacterial folate and a folate-related compound pteroate stimulate *C. elegans* germ cell proliferation through a folate receptor homolog FOLR-1-dependent pathway (Chaudhari et al., 2016). Besides, vitamin B2 (riboflavin) is a coenzyme precursor to regulate redox reactions. A study has suggested that heat-killed *E. coli* OP50 is poor quality food and does not support *C. elegans* development. However, adding extra vitamin B2 or a trace amount of live *E. coli* OP50 supports the growth of *C. elegans* fed on heat-killed OP50, suggesting that intestinal microbiota provide essential micronutrients such as vitamin B2 (Qi et al., 2017). Vitamin B12 deficiency has been linked to developmental disorders and metabolic abnormalities associated with various phenotypes that include decreased fertility and growth retardation (Bito et al., 2013). Vitamin B12 provided by *Comamonas aquatica* is an indispensable cofactor for detoxification and metabolite biosynthesis, which has been shown to accelerate development in *C. elegans via* the methionine/S-Adenosylmethionine (SAM) cycle (Watson et al., 2014). Additionally, vitamin B1 (thiamine) and its dephosphorylated form thiamine pyrophosphate (TPP) is the key regulator in biomacromolecules (nucleotides and amino acids) biosynthesis, and microbiota provides thiamine to its host to support host growth in *Drosophila* (Sannino et al., 2018). Aside from providing vitamins directly to the host, some bacteria contain complementary biosynthetic pathways that maintain host growth. A recent research suggests that *Acetobacter pomorum* and *Lactobacillus plantarum* modify the nutritional environment of *Drosophila* by providing intermediates of the missing micronutrients, such as biotin and pantothenate, therefore promoting the growth of juveniles (Consuegra et al., 2020). Consequently, as exogenous signals, bacterial derived vitamins stimulate animal germ cell proliferation and support animal growth.

### 2.2. Iron related metabolites

Iron is a cofactor essential for multiple fundamental cellular processes, including oxygen transport, energy metabolism, and DNA synthesis (Wang and Pantopoulos, 2011). To maintain an optimal physiological iron level, the expression levels and activities of iron carriers, iron transporters, and iron regulatory and storage proteins have to be tightly regulated (Gao et al., 2019). In recent years, iron metabolism has emerged as a critical regulator of cooperative host–microbe interaction (Chen and Ayres, 2020). Due to its rapid development, *C. elegans* is ideally suited for studying the mechanisms by which microbial metabolites affect animal development at a large scale and through high-throughput techniques. Based on a high-throughput screening of a single gene mutant library, a recent study has shown that reducing levels of bioavailable bacterial iron by feeding wild-type *E. coli* associated with iron chelators to *C. elegans* can delay development (Zhang et al., 2019). In contrast, supplementing 4 mM iron to *C. elegans* that are fed with an *E. coli fepG* mutant, an iron transporter deficient strain that is a poor iron source for *C. elegans*, can fully rescue the *C. elegans* developmental delay (Zhang et al., 2019). This suggests that *C. elegans* needs bacteria with proper levels of iron to support optimal animal development (Zhang et al., 2019).

Siderophores are chemically diverse group of secondary metabolites required for almost all bacterial species to obtain iron for metabolism (Boiteau et al., 2016). Diffusible siderophores secreted by bacteria can interact with their host in cooperative, exploitative and competitive ways (Kramer et al., 2020). Remarkably, enterobactin (Ent), a siderophore produced by the co-cultured bacterium *E. coli*, binds to *C. elegans* ATP synthase ɑ subunit to facilitate mitochondrial iron uptake, thereby supporting the growth of *C. elegans* (Qi and Han, 2018). In contrast to *C. elegans*, Ent acts as a virulence factor in mouse and human neutrophils; Ent inhibits ROS generation and neutrophil extracellular trap formation without altering neutrophil polarization and chemotaxis, suggesting that it may act as an anti-radical defense system that neutralizes neutrophil anti-microbial immunity in mouse and human models (Saha et al., 2017). Thus, the iron tug-of-war between host and commensal bacteria highlights the important role bacterial metabolites play in host iron homeostasis and development.

### 2.3. Lipids

Lipid metabolism is conserved among different organisms and affects all aspects of biology (Watts and Ristow, 2017). However, in higher animals, determining how microbial-derived lipids affect growth is challenging to study. Developmental time-courses can be long and disentangling the microbiota’s lipid contributions from other diet sources is difficult. Since *C. elegans* is a bacterivore, the microbiota diet can be precisely controlled. Furthermore, its short lifespan and hermaphroditic self-reproduction allow researchers to investigate the lipid composition from individual bacterial source with rapid high throughput.

For *C. elegans*, multiple lipids including palmitoleate, vaccenate, and cyclopropane fatty acids (CFAs) are predominantly acquired from dietary bacteria (Satouchi et al., 1993; Perez and Van Gilst, 2008). These lipids have various effects during different *C. elegans* developmental stages. Palmitoleate and cis-vaccenate are the only two unsaturated fatty acids in *E. coli*. Together they make up the bulk of the fatty acids in *E. coli* membrane, and account for around half of the total lipids content (Magnuson et al., 1993). It is known that *str-2* encodes a chemosensory G protein-coupled receptor (GPCR) that regulates transcription of key lipid metabolism enzymes (Dixit et al., 2020). Supplementation of palmitoleate and oleate (a naturally occurring fatty acid in animals and plants) in addition to the standard OP50 diet, increases the life span of both wild type and *str-2* mutant *C. elegans* (Dixit et al., 2020). This is an intriguing finding, since supplementation of oleate alone is not able to increase the life span of the *str-2* mutant. Palmitoleate is required to restore the life span reduction caused by the lack of STR-2 (Dixit et al., 2020). In addition to lengthening *C. elegans* adult aging, bacterial lipids, such as cyclopropane fatty acids, can also prolong the duration that *C. elegans* remain as a dauer larva, a facultative developmentally arrested larval stage. CFAs are synthesized by the enzyme cyclopropane fatty acyl phospholipid synthase (*cfa*) from monounsaturated fatty acids (MUFAs) during the stationary phase of bacterial growth, as part of the stringent response. The formation of CFA is thought to help stationary phase bacteria cope with increased acidity due to acetate accumulation; however, its presence can delay dauer recovery to adult development (Chang and Cronan, 1999; Kaul et al., 2014; Orr et al., 2019). Reduced level of CFAs and its resulting reciprocal increase in MUFAs have been shown to enhance the dauer recovery through chemosensation (Kaul et al., 2014). The enhanced recovery requires Guanylate Cyclase *daf-11* and the insulin peptide *ins-6* in sensory neurons (Kaul et al., 2014). This suggests that dauer larva can assess energetic value of stationary phase- versus dividing bacteria in the environment. By sensing the presence of specific fatty acids, developmentally arrested dauer larva can decide whether sufficient nutrients are available to exit the dauer stage and proceed with reproductive development (Kaul et al., 2014).

Short chain fatty acids (SCFAs) are fatty acids that have 6 or fewer carbons, and they are mainly produced through microbial fermentation. Butyrate is known to be a histone deacetylase inhibitor and regulates gene transcription levels in animals (Koh et al., 2016). Increased concentrations of butyrate, acetate, and propionate affect energy metabolism and induce reduction in fat deposition in *C. elegans*, potentially through epigenetic factors involving histone deacetylation (Zheng et al., 2010; Zheng and Greenway, 2012). In addition, butyrate-producing microbes can protect *C. elegans* against protein folding disruption from co-cultured pathogens (Walker et al., 2021). Microbial SCFAs have also been shown to regulate lipid and carbohydrate metabolism through recognition of SCFAs from enteroendocrine cells to maintain host intestinal stem cells in *Drosophila* (Neophytou and Pitsouli, 2022). Collectively, the evidence above shows that lipids derived from bacteria are crucial for the host to sustain its lifespan, recover from developmental arrest, and maintain its fat deposition level.

### 2.4. Bile acids

Microbial bile acids are important hormones that modulate host cholesterol metabolism and energy balance through nuclear receptors and G-protein-coupled receptors (Wahlström et al., 2016). Aerobic degradation of bile acids has primarily been studied in soil bacteria such as *Pseudomonas* and *Comamonas* (Barrientos et al., 2015; Horinouchi et al., 2019; Feller et al., 2021). The bacterivore *C. elegans* can grow and reproduce on a variety of bacterial diets. This makes *C. elegans* uniquely suited for studying the effects of bacterial bile acids on host. Androstadienediones (ADDs) is a steroid compound derived from bacterial bile acid degradation, it negatively affects the reproduction rate and developmental speed in *C. elegans* (Mendelski et al., 2019). Bacterial metabolites deoxycholic acid (DCA) and lithocholic acid (LCA) are two major secondary bile acids derived from primary bile acids (Ridlon et al., 2006). Recent studies have shown that bacterial 3β-hydroxydeoxycholic acid (isoDCA) promotes generation of colonic peripheral regulatory T cells, contributing to the maintenance of host immunological homeostasis and intestinal health (Campbell et al., 2020; Song et al., 2020). In macrophages and monocytes, bile acids derived from commensal bacteria regulate the anti-inflammatory response of immune cells *via* the G protein-coupled bile acid receptor 1 and the nuclear receptor (Fiorucci et al., 2009; Brestoff and Artis, 2013). Moreover, increased levels of DCA and LCA in the blood, bile, and feces contribute to disease processes, including colon and liver cancers (Ou et al., 2013; Yoshimoto et al., 2013). In contrast, exogenous supplementation with LCA prolongs the lifespan of *Drosophila* (Staats et al., 2018). Taking together, secondary bile acids appear to be a double-edged sword, extending lifespan while increasing cancer risk.

### 2.5. Tryptophan and tryptophan-derivatives

Tryptophan is crucial for systemic homeostasis since it is involved in essential pathways that regulate nutrient sensing, metabolic stress response, and immunity (Gostner et al., 2020). Tryptophan concentration can be modulated by animal diet as well as its microbiota (Gostner et al., 2020). How bacterial tryptophan affects *C. elegans* development and physiology helps with understanding tryptophan’s role in maintaining the homeostasis. For both humans and *C. elegans,* tryptophan is an essential amino acid that can only be supplied by food. Tryptophan and its derivatives have recently drawn attention for their effects in many physiological processes. Dietary tryptophan suppresses toxicity of α-Synuclein, an aggregation-prone protein that is involved in Parkinson’s disease and other synucleinopathies, and delays aging in *C. elegans* (Van Der Goot et al., 2012). Tryptophan can be digested by microbes to produce indole *via* Tryptophanase (TnaA). Health span is the length of time an individual remains healthy and free of age-related infirmities; indole from commensal bacteria has been shown to extend health span in multiple organisms including *C. elegans* and *D. melanogaster* (Sonowal et al., 2017). For example, the health span in *C. elegans* is often characterized by behavioral activities such as pharyngeal pumping rate and locomotor thrashing frequency. Mediated by aryl hydrocarbon receptor (AHR), indole can alter gene expression levels of aged *C. elegans* for ~500 genes, including those that are responsible for neurogenesis and DNA replication, so that the expression profile approximates those of young, healthy animals (Edwards et al., 2015; Sonowal et al., 2017; Brinkmann et al., 2021). Besides aging and health span, tryptophan is also involved in development of the reproductive system. Dietary tryptophan metabolized by bacteria rescued sterility caused by the absence of the nuclear receptor *nhr-114* in *C. elegans* (Gracida and Eckmann, 2013). Additionally, microbial tryptophan metabolites indole-3-ethanol, indole-3-pyruvate, and indole-3-aldehyde regulate gut barrier function *via* the aryl hydrocarbon receptor and protect the host from inflammation in a mouse model of colitis (Scott et al., 2020). However, tryptophan is not always beneficial in micro-host interactions. Enteropathogenic *E*. *coli* (EPEC) also requires tryptophan and TnaA to induce paralysis and subsequent death in *C. elegans* (Anyanful et al., 2005). Tryptophan and its derivatives from bacteria can therefore be beneficial to animals by extending their health span, but it can also make animals vulnerable to pathogens.

### 2.6. Peptidoglycan metabolites

Peptidoglycan (PG) is an essential and distinctive component of the bacterial cell wall. It resides outside of the cytoplasmic membrane for both Gram-positive and Gram-negative bacteria (Neidhardt, 1996). During bacterial proliferation, PG is hydrolyzed into fragments such as muropeptide (Irazoki et al., 2019). As a result of the recognition of muropeptides, bacteria modulate their metabolism, cell division, and cell wall homeostasis to sense and respond to the host environment and antibiotic stress (Pensinger et al., 2018). PG degraded metabolites can affect host physiology *via* a variety of pathways (Wheeler et al., 2014). In eukaryotes, peptidoglycan recognition proteins (PGRPs) or Nod-like receptors (NLRs) are required for recognizing intact or fragmented PG of the pathogen to initiate immune responses (Girardin et al., 2003; Royet et al., 2011). In an *in vitro* study, muramyl dipeptide (MDP), the minimal essential motif of PG, was found to be responsible for immunoadjuvant activity and synergistically enhance osteoclast formation through nucleotide-binding oligomerization domain 2-mediated signaling (Yang et al., 2005). Moreover, MDP can be digested by macrophages, and induce slow-wave sleep responses mediated by immune effectors (Krueger et al., 1984).

Besides the role in pathogenicity and immune response, PG fragments produced by *E. coli* have a beneficial role in regulating host development (Tian and Han, 2022). Due to its easy manipulation and well-defined microbiota, *C. elegans* is an excellent model for investigating the effects of bacterial metabolites on host physiology. Using the *E. coli* Keio Collection and a sub-library that contains 57 PG metabolism mutants, Krueger et al. identified 4 PG mutant bacterial strains that when fed to *C. elegans*, significantly delay nematode development (Tian and Han, 2022). Additionally, PG metabolites disaccharide muropeptides were found to act as an ATP synthase agonist by entering the animal’s mitochondria and binding to ATP synthase to promote energy production, thus improving mitochondria homeostasis and development in *C. elegans* (Tian and Han, 2022). Collectively, PG metabolites play important roles in host physiology, from stimulating immune response to enhancing energy production, which reflects an adaptive interaction between bacteria and their hosts.

## 3. Methods for metabolites identification in regulating host development and physiology

**Figure 2:**
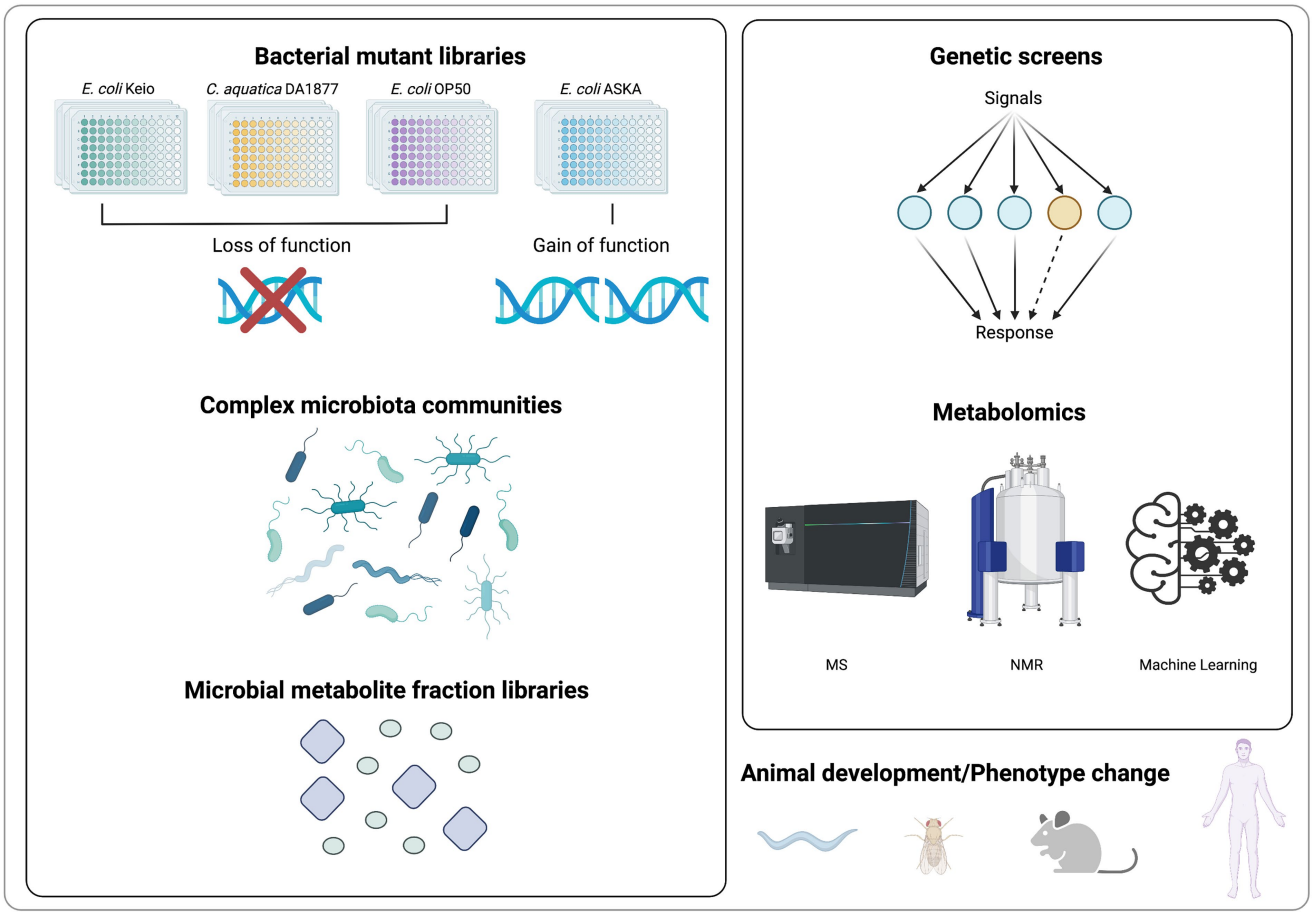
Screening methods for metabolites identification in regulating host development and physiology.

Due to the high degree of crosstalk within and between kingdoms, studying host-microbiota interactions remains challenging. As breakthrough studies, multiple bioactive microbial targets have been identified by metabolite-focused research. Recent advances in high-throughput experimental and analytical methods have made it possible to identify functional metabolites in fundamental development regulation. In this section, we summarize methods for identifying microbial metabolites in regulating host development and physiology (Figure 2).

### 3.1. Genetic screen

A genetic screen of bacterial mutants is an effective approach to discover molecular factors that promote animal development and physiology. In order to identify bacterial metabolites that are required for developmental or physiological processes, a variety of mutational gene deletion or transposon insertion-based bacterial libraries have been generated and fed to *C. elegans* (García-González et al., 2017). If the fed mutant bacteria affects nematode behavior, development, or lifespan, then potential metabolite function can be inferred from known bacterial metabolic networks, and confirmed with direct supplementation to the worm’s diet (Zhang et al., 2017). In contrast to conventional pharmacological screening, the use of *C. elegans* and bacterial mutant library enables the functional analysis of uncharacterized or poorly characterized molecules synthesized in physiologically relevant amounts from the co-cultured bacteria.

The Keio *E. coli* BW25113 collection is the most widely used bacterial mutant library. It contains 3,985 in-frame, single-gene deletion mutants, which covers its 93% annotated genes (Baba et al., 2006). As previously mentioned, a variety of metabolites related to fundamental development were first identified through the high-throughput genetic screens in *C. elegans*, such as PG muropeptides and enterobactin (Qi and Han, 2018; Tian and Han, 2022). Moreover, a mutation library, containing ~2000 mutations in the background of the standard laboratory *C. elegans* food source *E. coli* OP50, has also been used to screen for microbial gene activities necessary for *C. elegans* growth and development (Govindan et al., 2015). Complementing loss-of-function libraries, the gain-of-function library *E. coli* ASKA is also widely employed for understanding the metabolic links in the regulation of host physiology (Kitagawa et al., 2005; Pryor et al., 2019). In addition to laboratory *E. coli*, a variety of soil bacteria, including *Comamonas,* have been studied as nutrition sources for *C. elegans* (Dirksen et al., 2016). Since altering bacterial diets can have a profound effect on worm physiology, a mutant strain collection based on *Comamonas aq.* DA1877 was created by transposon-based mutagenesis. It contains 5,760 mutations, and has been used to explore new complex interactions between bacteria, metabolic regulation, and physiology (Watson et al., 2013). By applying a multi-species systems approach to the mutant library strategy, vitamin B12 from fed *Comamonas aq.* was identified to accelerate *C. elegans* development (Watson et al., 2014).

### 3.2. Comparative metabolomics

Comparative mass spectrometry (MS)-based metabolomics is a strategy for identification of microbial metabolites in response to diet and endogenous host factors changes (Covington et al., 2017). It identifies microbial metabolites related to fundamental animal developmental processes by comparing metabolomic data from different biological sources, such as wild-type microbiome versus developmental-disordered microbiome. For example, a metabolomic analysis revealed that methionine deficiency in bacterial medium decreases the production of bacterial metabolites that are essential for phosphatidylcholine synthesis in *C. elegans* (Lin and Wang, 2017). Using comparative metabolomics, researchers found that the levels of free amino acids and volatile organic compounds from fecal samples are associated with children Pervasive Developmental Disorder Not Otherwise Specified (PDD-NOS; De Angelis et al., 2013). Comprehensive comparison of the metabolic profiles from intestinal luminal biofluid highlighted the function of basic amino acids and specific polyamines in healthy gut microbiota (Matsumoto et al., 2012).

In addition to MS, nuclear magnetic resonance (NMR) spectroscopy is another principal analytical technique used in metabolomics. NMR spectroscopy is nondestructive and nonbiased, requiring less or no sample treatment or chemical derivatization. As it is highly automatable and makes high-throughput, large scale metabolomics more feasible in identification of novel compounds (Emwas et al., 2019; Letertre et al., 2020). Using NMR-based metabolomic approaches, gut microbiota-derived metabolites such as hippuric acid and trigonelline are identified in an obesity-associated metabolic phenotype (Calvani et al., 2005). In recent years, the integration of metabolomics analysis with machine learning and artificial intelligence, which allow for an explicitly learning and relationship prediction in metabolomic data processing, will enable more applications of MS-based metabolomics in metabolite identification (Sen et al., 2020). Additionally, with the development of single-cell metabolomics (Duncan et al., 2019), it is now feasible to explore the function of metabolites at single cell level. For example, single-cell metabolomics has uncovered differential metabolites including arginine, glutamine, and glutamic acid between individual wild type and chemo resistant cells (Xu et al., 2021). Those approaches will clearly aid the discovery of novel microbial metabolites in regulating animal development and physiology.

## 4. Conclusion and perspective

Research over the past decade has shown that bacterial microbiota influences numerous physiological aspects in the host, including germ cell proliferation, immune system activation, growth and reproduction, energy metabolism, intestinal health, longevity, and cancer. Microbial metabolites are considered a key part for maintaining host physiological homeostasis. It is now clear that microbial metabolites such as vitamins, iron, lipids, bile acids, tryptophan and tryptophan-derivatives, PG metabolites, play an important role in maintaining animal development and overall fitness. In this review, we summarized how bacterial metabolites may interact with host metabolic pathways to regulate host life cycles. In addition to providing functional building blocks for the host, microbial metabolites are a more informative means of cooperation between bacteria and their host. Through conserved metabolic networks, such as insulin/IGF-1 and steroid hormone systems, they are sensed by the host, modulating digestion and nutrient flux, thereby regulating worm development and physiology. As a popular choice of animal model for microbe-host interaction studies, *C. elegans* has a completely sequenced genome and benefits from the increasing availability of tools for analyzing basic cellular processes. The genetic manipulation of animals and microbes, coupled with analysis of transcription, metabolism, and physiology of hosts and microbes, has provided definitive insights into the genetic and molecular basis of how the gut microbiota influences host growth and physiology. It will continue to play an important role in elucidating nutrient functions and metabolic networks.

Although many metabolites can be obtained from bacteria, the presence of specific transporters can play pivotal roles for gut microbes to regulate the availability of these metabolites during homeostasis, which still need to be elucidated. Also, a broader spectrum of abundant microbial metabolites from diverse organisms still to be identified, and the paucity of genetic tools for engineering different organisms is limiting new bacteria-host interaction research areas. From the host perspective, how they rely on microbial metabolites to sense the state of nutrient availability and the underlying mechanism remains to be investigated. Future research will also require understanding whether bacterial metabolites that effect animal development can also therapeutically ameliorate human diseases caused by nutrient deficiencies. Overall, identification of microbial metabolites involved in animal development and physiological changes will enable better understanding of the fundamental metabolic processes within the context of microbe-host interactions. Additionally, it could potentially enable targeted therapies that manipulate the production of specific microbial metabolites to improve our overall health.

## Author contributions

MF and BG drafted the original manuscript. LG and QS revised the manuscript. All authors contributed to the article and approved the submitted version.

## Funding

This work was supported by the Texas A&M Startup grant from TEES and Department of Chemical Engineering to QS.

## Conflict of interest

The authors declare that the research was conducted in the absence of any commercial or financial relationships that could be construed as a potential conflict of interest.

## Publisher’s note

All claims expressed in this article are solely those of the authors and do not necessarily represent those of their affiliated organizations, or those of the publisher, the editors and the reviewers. Any product that may be evaluated in this article, or claim that may be made by its manufacturer, is not guaranteed or endorsed by the publisher.
